# Deep Learning Using Endobronchial-Ultrasound-Guided Transbronchial Needle Aspiration Image to Improve the Overall Diagnostic Yield of Sampling Mediastinal Lymphadenopathy

**DOI:** 10.3390/diagnostics12092234

**Published:** 2022-09-16

**Authors:** Ching-Wei Wang, Muhammad-Adil Khalil, Yi-Jia Lin, Yu-Ching Lee, Tsai-Wang Huang, Tai-Kuang Chao

**Affiliations:** 1 Graduate Institute of Biomedical Engineering, National Taiwan University of Science and Technology, Taipei 106335, Taiwan; 2 Graduate Institute of Applied Science and Technology, National Taiwan University of Science and Technology, Taipei 106335, Taiwan; 3 Department of Pathology, Tri-Service General Hospital, Taipei 11490, Taiwan; 4 Institute of Pathology and Parasitology, National Defense Medical Center, Taipei 11490, Taiwan; 5 Department of Thoracic Surgery, Tri-Service General Hospital, National Defense Medical Center, Taipei 11490, Taiwan

**Keywords:** endobronchial-ultrasound-guided transbronchial needle aspiration, lung cancer, whole-slide images, deep learning

## Abstract

Lung cancer is the biggest cause of cancer-related death worldwide. An accurate nodal staging is critical for the determination of treatment strategy for lung cancer patients. Endobronchial-ultrasound-guided transbronchial needle aspiration (EBUS-TBNA) has revolutionized the field of pulmonology and is considered to be extremely sensitive, specific, and secure for lung cancer staging through rapid on-site evaluation (ROSE), but manual visual inspection on the entire slide of EBUS smears is challenging, time consuming, and worse, subjective, on a large interobserver scale. To satisfy ROSE’s needs, a rapid, automated, and accurate diagnosis system using EBUS-TBNA whole-slide images (WSIs) is highly desired to improve diagnosis accuracy and speed, minimize workload and labor costs, and ensure reproducibility. We present a fast, efficient, and fully automatic deep-convolutional-neural-network-based system for advanced lung cancer staging on gigapixel EBUS-TBNA cytological WSIs. Each WSI was converted into a patch-based hierarchical structure and examined by the proposed deep convolutional neural network, generating the segmentation of metastatic lesions in EBUS-TBNA WSIs. To the best of the authors’ knowledge, this is the first research on fully automated enlarged mediastinal lymph node analysis using EBUS-TBNA cytological WSIs. We evaluated the robustness of the proposed framework on a dataset of 122 WSIs, and the proposed method achieved a high precision of 93.4%, sensitivity of 89.8%, DSC of 82.2%, and IoU of 83.2% for the first experiment (37.7% training and 62.3% testing) and a high precision of 91.8 ± 1.2, sensitivity of 96.3 ± 0.8, DSC of 94.0 ± 1.0, and IoU of 88.7 ± 1.8 for the second experiment using a three-fold cross-validation, respectively. Furthermore, the proposed method significantly outperformed the three state-of-the-art baseline models, including U-Net, SegNet, and FCN, in terms of precision, sensitivity, DSC, and Jaccard index, based on Fisher’s least significant difference (LSD) test (p<0.001). For a computational time comparison on a WSI, the proposed method was 2.5 times faster than U-Net, 2.3 times faster than SegNet, and 3.4 times faster than FCN, using a single GeForce GTX 1080 Ti, respectively. With its high precision and sensitivity, the proposed method demonstrated that it manifested the potential to reduce the workload of pathologists in their routine clinical practice.

## 1. Introduction

In normal physical examinations, one of the most difficult malignancies to identify at an early stage is lung cancer [1], which is one of the most often diagnosed diseases. As the number of lung cancer patients rises, the health care systems in both industrialized and developing nations are being put under immense strain [2]. Lung cancer is mainly divided into non-small-cell lung cancer (NSCLC) and small-cell lung carcinoma. About 80% to 85% of lung cancers cases are NSCLC. The subtypes of NSCLC are adenocarcinoma, squamous-cell carcinoma, and some other subtypes [3]. Although there have been new developments in the diagnosis, classification, and treatment of lung cancer, the overall survival rate is still poor [4]. At the time of diagnosis, the majority of the cases have distant metastasis [5]. Because of their advanced clinical stage, the great majority of patients with NSCLC do not need surgical resection [6]. Mediastinal lymphadenopathy (ML) presents a diagnostic challenge. ML may be caused by infections, granulomatous disease, reactive hyperplasia, and metastatic tumor [7]. In patients with NSCLC, determining mediastinal and hilar metastases may help guide therapy decisions, provide prognostic information, and help with patient care [8]. ML nodes need to be sampled to reach a diagnostic conclusion. Currently, there are several evaluation methods, such as positron emission tomography/computed tomography (PET/CT), endobronchial ultrasound (EBUS)-guided transbronchial needle aspiration (TBNA), B-mode morphological ultrasound, and elastography, that can be used to classify the nodal stage before surgery [9]. Although PET/CT is the main instrument for the preoperative examination of lung cancer patients, EBUS-TBNA has superior test performance and PET/CT cannot be regarded as an alternative method to be used to replace EBUS-TBNA for tissue sampling [10].

EBUS-TBNA is a new clinical technology and currently the preferred method of assessing advanced lung cancer with mediastinal lymphadenopathy. Real-time ultrasound guidance gives good diagnostic value during mediastinal lymph node collection with EBUS-TBNA, a minimally invasive method. Some of its benefits include being cost-effective, easy to use, and safe [11]. Because of this, EBUS-TBNA is considered a better option for mediastinal lymph node sampling than traditional mediastinoscopy. When combined with EBUS-TBNA, EBUS-TBNA may provide clinical information in 19% of patients, negating the need for further invasive testing. Direct smears from needle aspiration and brushing specimens allow the EBUS-TBNA to be used for on-site assessment [11,12,13,14,15]. The EBUS-TBNA treatment, which requires only little sedation and may be performed as an outpatient procedure, can be used to get a sample from the hilar lymph nodes [16]. There have been several prospective protocol-based studies [17] that have shown that EBUS-TBNA is 95 percent accurate in detecting and staging lung cancer. Microscopic pathological examination is usually the gold standard for diagnosing different types of cancer [18,19]. When dealing with suspected metastatic mediastinal lesions, rapid on-site assessment (ROSE) seems to be especially helpful in improving the EBUS-diagnostic TBNA’s yield [20]. The preliminary diagnosis provided by ROSE may minimize the number of invasive procedures (such as mediastinoscopy) required in the future [21]. When used in clinical settings, the ROSE of EBUS-TBNA needs the collaboration of many medical specialists for analysis and diagnosis, which is not always achievable owing to a lack of medical personnel. Furthermore, if malignant cells are missed during the manual screening procedure, the patient runs the risk of undergoing unneeded surgery as a result of their condition worsening.

It is now possible to convert glass slides into whole-slide images (WSIs), which enables the examination of pathological images using computer-based algorithms [22]. WSIs are high-resolution images with huge file sizes on the order of 10 gigapixels, making it difficult for pathologists to manually examine all the information on the histopathology slide due to the large quantity of information on each slide. Thus, a qualified cancer diagnosis necessitates peer review and consensus, a requirement that can be costly to meet in hospitals and small cancer centers with a scarcity of skilled pathologists. Deep learning algorithms have been frequently used in pathological image analysis applications in recent years, demonstrating their strength in representation learning. Automating the pathological image analysis aids pathologist in making accurate diagnoses in a short amount of time. Deep learning has previously shown promise in helping pathologists diagnose, classify, and segment cancer [23,24,25]. Examples include Rijthoven et al. [26], who proposed a segmentation model that combined context and details by utilizing many branches of encoder–decoder convolution neural networks for breast and lung cancer segmentation in WSIs. Wang et al. [27] proposed a fast and fully automatic hierarchical deep learning approach that utilized the coarse-to-fine strategy for bone marrow nucleated differential count on WSIs. Dov et al. [28] proposed a weakly supervised two-stage deep learning model for detecting thyroid cancer on WSIs. Tang et al. [29] proposed a DeFusionNet that can efficiently and accurately detect defocus blur. Masud et al. [30] utilized pretrained deep learning models for the detection of breast cancer in ultrasound images.

**Figure 1:**
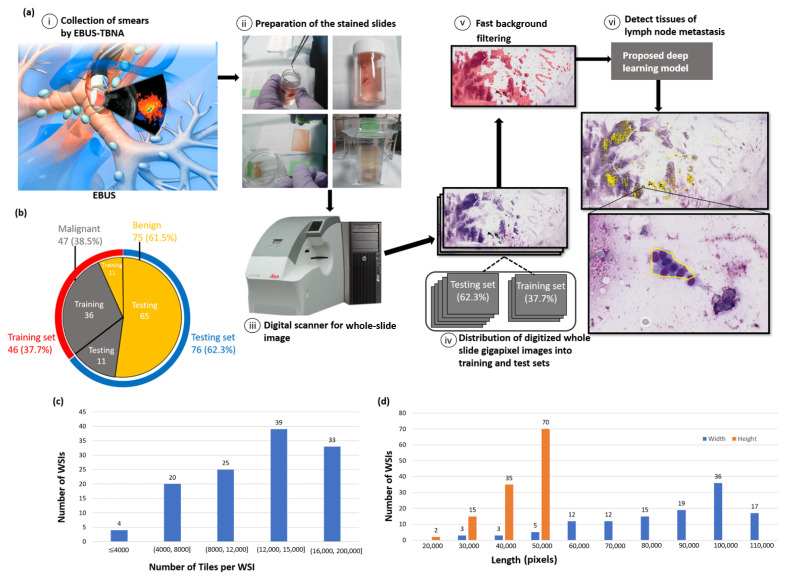
System workflow and dataset information. (**a**) System workflow; (**a**, i) collection of patients’ lung smear samples through EBUS-TBNA; (**a**, ii) preparation and staining of EBUS smears slides using Liu’s staining; (**a**, iii) digitalization of Liu stained microscopic slides at the microscopic resolution (20× magnification); (**a**, iv) random distribution of digitized whole-slide gigapixel images into a separate training (37.7%) set and a testing (62.3%) set for the first experiment and a three-fold cross validation for the second experiment; (**a**, v) processing of WSIs with fast background filtering; (**a**, vi) rapid detection of enlarged mediastinal lymph nodes metastasis in EBUS-TBNA cytological slides using the proposed deep learning model in seconds. (**b**) Distribution of dataset for each class, as well as for training and testing sets for the first experiment and a three-fold cross validation for the second experiment. (**c**) Distribution of the number of tiles per WSI. (**d**) Size distribution of the WSIs, with width and height represented in blue and orange, respectively.

To the best of the authors’ knowledge, this is the first research on automatic segmentation of enlarged mediastinal lymph nodes metastasis in EBUS-TBNA cytological slides. The proposed automatic deep learning system is shown to be capable of detecting metastases of enlarged mediastinal lymph nodes and immediately notifying clinical doctors as a guide for subsequent adequate treatment. Figure 1 presents the workflow of the system and information on the dataset in detail. In a quantitative evaluation on the segmentation performance, the proposed method is compared with three state-of-the-art deep learning models, including U-Net [31], SegNet [32], and FCN [33], since this is the first research on automatic segmentation of lymph nodes metastasis in EBUS-TBNA cytological slides.

## 2. Methods

**Figure 2:**
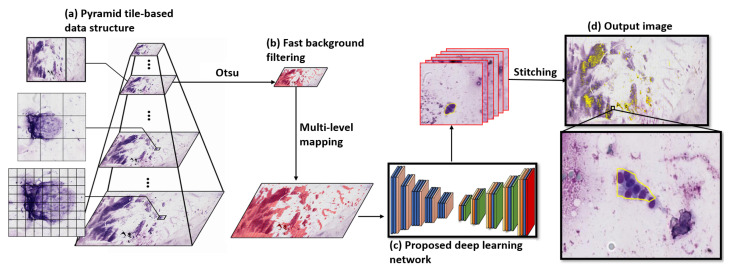
The overview of the proposed deep learning analysis system. (**a**) Each WSI is converted into a patch-based hierarchical structure (each tile is represented by a black rectangular box). (**b**) Each WSI is processed using the fast background filtering model to efficiently discard all the background. (**c**) Each tile is examined by the proposed deep convolutional neural network for segmentation of metastatic lesion from EBUS-TBNA WSIs. (**d**) The tiles are then stitched together to obtain an output image.

In this study, we developed a fast, efficient, and fully automatic deep-convolutional-neural-network-based AI system to segment metastatic lesions in EBUS-TBNA cytological WSIs. Figure 2 presents the overview of the deep-convolutional-neural-network-based AI system. Each WSI was converted into a patch-based hierarchical structure and processed using the fast background filtering model and the proposed deep convolutional neural network for the segmentation of a metastatic lesion from EBUS-TBNA WSIs. Figure 4 illustrates the detailed architecture of the proposed deep learning model.

### 2.1. Whole-Slide Image Processing

Figure 2 shows the framework for the segmentation of metastatic lesion from EBUS-TBNA WSIs. For the efficiency of the data assessment, WSIs tend to be encoded and stored in a pyramid structure, that contains several layers of images at different magnification rate, by various microscopic scanners such as Leica, Hamamatsu, and Philips. In order to deal with gigapixel WSIs effectively, each WSI I(i,j) was converted into a patch-based structure $H={\left\{h_{\alpha ,\beta }^{v}\left(x,y\right)\right\}}_{v=1}^{N}\in I\left(i,j\right)$, where α, β, *x*, *y*, and *v* represent the patch column index, patch row index, patch horizontal subindex, patch vertical subindex, and the image level, respectively. When v=N, α, β, *x*, and *y* were calculated as follows:

(1) $$\begin{array}{cc} \alpha =\lfloor i/w\rfloor , & \beta =\lfloor j/\psi \rfloor ,\\ x=i-\alpha \times w, & y=j-\beta \times \psi \end{array}$$

where *w* and ψ denote the patch width and patch height, respectively. The values α,β,x, and *y* are in the range {0,⋯,γ−1}, {0,⋯,ζ−1}, {0,⋯,w−1}, and {0,⋯,ψ−1}, respectively; (w,ψ) = (512,512) in this study. The ⌊⌋ operation was devised for rapid processing of WSI by discarding the border part of a slide, which tends to have a low probability of containing regions of interest.

Firstly, a WSI was processed with fast background filtering by Otsu’s method applied onto $h_{\alpha ,\beta }^{z}\left(x,y\right)$ at the level closest to and greater than or equal to the size of a unit tile and then mapping the filtered image back to the highest level to efficiently discard all the background patches (patches that contains less than 70% tissue samples), significantly decreasing the computational cost per slide. The value of z is formulated in Equation (2). As the dimensionality of WSIs was tremendous, fast background filtering was applied in order to efficiently discard the background areas which contained no cells and rapidly decreased the dimension. For further analysis, previous studies [34,35] also showed that Otsu’s method performed well to remove the background regions of a WSI on the slide’s thumbnail image to efficiently discard all background tiles, thus drastically reducing the amount of computation.

(2) $$z=argmin_{v}\left(\gamma \times \zeta \geq 1\wedge card\left(h^{v}\right)\geq w\times \psi \right)$$

The proposed deep convolutional neural network *L* was applied for a fast WSI analysis. The proposed deep convolutional neural network is described in detail in the next section. Each tile of $h_{\alpha ,\beta }^{N}\left(x,y\right)$ was analyzed by the proposed convolution model *L* to generate the probabilities of malignant cells as shown in Equation (3).

(3) $$p_{\alpha ,\beta }^{N}{\left(x,y\right)}^{c}=L\left(h_{\alpha ,\beta }^{N}\left(x,y\right)\right)$$

where c=0,⋯,C represents the number of types of tissue to be identified, and 0, 1 and 2 represent the background, the nontarget, and the target cell type, respectively.

A two-dimensional pixel-based class map was produced as the index of the cell type that had the maximum probability of the pixel, using Equation (4).

(4) $$m_{\alpha ,\beta }^{N}\left(x,y\right)=argmax_{c}\left(\left(p_{\alpha ,\beta }^{N}{\left(x,y\right)}^{c}\right)\right)$$

Then, the pixel-based segmentation results of tumor cells $R=\{r_{\alpha ,\beta }^{N}\left(x,y\right)\}$ were produced based on class map $m_{\alpha ,\beta }^{N}\left(x,y\right)$ using Equation (5). Equation (5) suppressed the nontumor information, producing the tumor information as the segmentation results.

(5) $$r_{\alpha ,\beta }^{N}\left(x,y\right)=\left\{\begin{array}{cc} I_{\alpha ,\beta }\left(x,y\right) & ,\;m_{\alpha ,\beta }^{N}\left(x,y\right)>1\\ \phi & ,otherwise \end{array}\right.$$

where ϕ represents a null set.

### 2.2. Proposed Convolution Network Architecture

**Figure 3:**
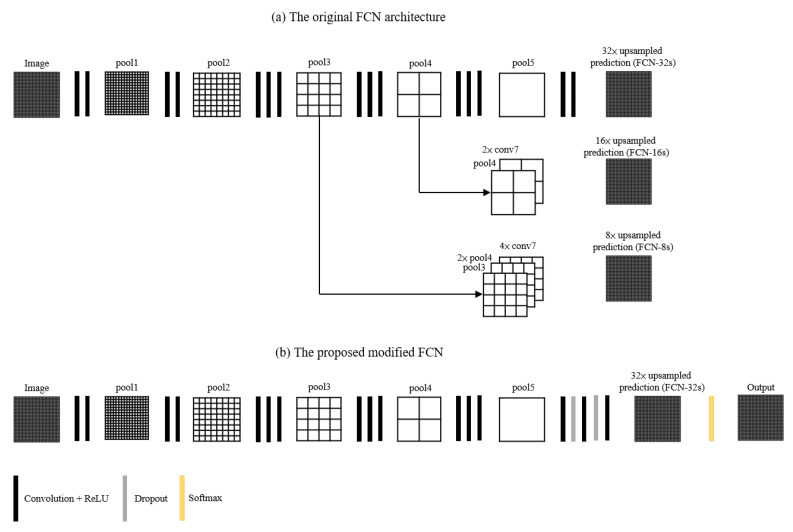
The comparison of the original FCN and the proposed modified FCN. (**a**) The architecture of original FCN. (**b**) The architecture of proposed modified FCN.

**Figure 4:**
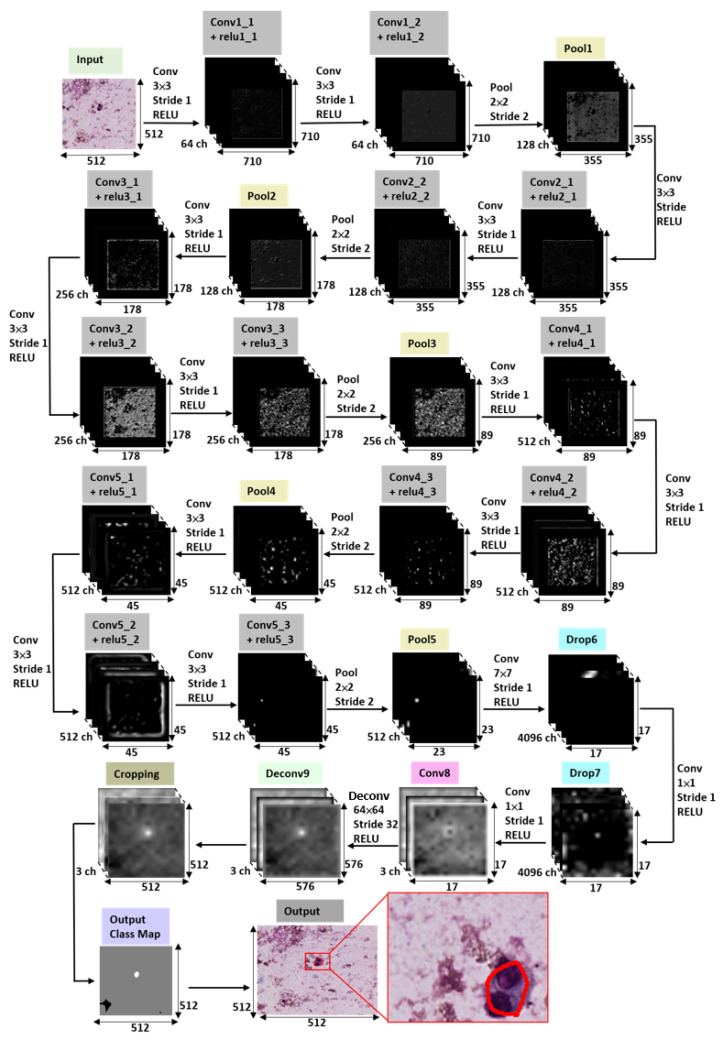
The detailed architecture of the proposed deep learning network.

A fully convolutional network (FCN) was introduced by Shelhamer et al. [33] and it was demonstrated to be successful in the tumor segmentation of breast cancer metastases [36], thyroid cancer [37], cervical cancer [38], and ovarian cancer [39]. Inspired by the fully convolutional network (FCN) framework of Shelhamer et al. [33], the proposed deep learning network architecture has two improvements, as shown in Figure 3. To begin, we employed a five-layer shallower architecture instead of the original seven-layer FCN architecture to deal with the issue of limited GPU memory in training. Secondly, we used a single-stream 32 s upsampling path instead of three upsampling paths to prevent overly fragmented segmentation results and to save computing time. The proposed deep learning network architecture consisted of six convolutional layers, five max-pooling layers, two dropout layers, one deconvolutional layer, and a cropping layer. Firstly, there were five convolutional layers, with the first two layers consisting of two convolution sequences (kernel size of 3×3 and stride size of 1, respectively) and the last three layers consisting of three convolution sequences (kernel size of 3×3 and stride size of 1, respectively), and a ReLU after each convolution layer. To downsample the feature maps, a max pooling layer (kernel size of 2×2 and stride size of 2) was added at the end of each convolution layer. After the five convolution layers and five max-pooling layers, there were two drop-out layers (dropout ratio of 0.5) with two convolutions (kernel size of 7×7 and stride size of 1 for the first convolution, and a kernel size of 1×1 and stride size of 1 for the second convolution, respectively), and a ReLU after each convolution. Following the two dropout layers was a convolution layer with a kernel size of 1×1 and a stride size of 1. Following the convolution layer, a deconvolution layer with kernel size of 64×64 and stride size of 32 was used to upsample the feature maps. Following the deconvolution layer, cropping was performed in order to match the input size. After cropping, softmax was employed to calculate the probability of each class. Finally, an argmax function was utilized to generate a two-dimensional pixel-based class map as the pixel’s index of the tissue type with the highest likelihood using Equation (4). The detailed architecture of the proposed deep learning network is shown in Figure 4 and Table 1.

## 3. Data and Results

### 3.1. Data Preparation

EBUS-TBNA cytology samples of patients were collected from the Department of Pathology at the Tri-Service General Hospital. This research was approved by the Institutional Review Board of the Tri-Service General Hospital (TSGH) (TSGHIRB No.1-107-05-171 and No.B202005070), and informed consent was formally waived by the approving committee. The data were deidentifed and used for a retrospective study without impacting patient care. All the methods were performed in accordance with the relevant guidelines and regulations. For this retrospective study, slides of nodal aspirates from consecutive EBUS-TBNA procedures performed at TSGH between January 2018 and December 2020 were obtained. We refer to two slides on average from a single lymph node as a case because each nodal sampling could involve more than one slide. All cases had an associated cell block that contributed to the final diagnosis. All the lung cancer patients had thoracic CT before EBUS-TBNA examination, which showed enlarged hilar or mediastinal lymph nodes. Each patient was aware of the indications, technique, and possible complications of EBUS-TBNA. EBUS-TBNA was performed under general endotracheal anesthesia and the procedure was performed only under moderate sedation. All the EBUS-TBNA procedures were performed by pulmonologists. On the advice of pulmonologists and thoracic surgeons, EBUS-TBNA was used to take selective lymph node samples.

There is still some limitation of EBUS-TBNA in mediastinal lymph nodes sampling. The size of the lymph node, the SUVmax of the lymph node, and the characteristics of the node in color Doppler image may provide information when the physician is performing the EBUS-TBNA procedure [40,41,42]. However, the yield rate of this procedure depends on the experience of the operator. The new diagnostic tool of elastography was applied to predict the lymph node status [43]. In this study, we analyzed the clinical data including the size of the lymph node, SUVmax of the tumor, and blue color proportion of the node in elastography (35%) to select the possible metastatic lymph nodes for EBUS-TBNA cytological examination.

Initially, aspirated material was pushed out by the internal stylet and smeared onto glass slides for immediate ROSE. Then, using the following staining procedures, a quick cytological Liu’s staining was carried out. The Liu A solution was added to alcohol-fixed endobronchial-ultrasound-guided transbronchial needle aspiration smears and allowed to stand for 30 s. The Liu B solution (about double the dose of A) was then added to the smears without discarding the Liu A solution. To stain for 2 min, the two solutions were completely mixed. Before microscopic analysis, the smears were rinsed with tap water and air dried or dried with filter paper. The EBUS-TBNA specimen’s cytological imaging data were evaluated. For histological assessment, the residual aspirate and additional needle routes were preserved in formalin solution. Staff pathologists examined cytology and tissue slides.

Based on the diagnosis by two cytopathologists, we classified the EBUS-TBNA samples into 47 positive slides from 24 patient cases and 75 negative slides from 38 patients. The positive group was defined as the cases of enlarged mediastinal lymph nodes accompanied by tumor cells metastasis, and the follow-up diagnosis of the positive cases was determined as adenocarcinoma (*n* = 20), squamous-cell carcinoma (*n* = 3), and small-cell carcinoma (*n* = 1). On the other hand, the negative group contained the cases diagnosed as having no evidence of tumor cells metastasis, and the associated follow-up diagnosis was determined as reactive lymph nodes (*n* = 36) and benign granulomatous lymphadenopathy (*n* = 2). Images were produced by a digital slide scanner (Leica AT Turbo (Leica, Germany) with a 20× objective lens and stored as a pyramid data structure with several downsampled variants of the base image in the same file encoded in the svs file format. This study employed a total of 122 slides, including 47 malignant slides and 75 benign slides. The average slide dimensions were 97,608 × 45,309 pixels with a physical size of 49.13×22.80 mm${}^{2}$. All the slides were anonymized during the data collection. Pixel-level annotations were generated as reference standards by two expert pathologists. Two experiments were performed in evaluation and the data division process in the first and second experiments was performed by random sampling of the slides. For the first experiment, the AI models were trained using a total of 46 WSIs, which accounted for 37.7% of the whole dataset and included 36 malignant slides and 10 benign slides, where around 0.02% benign cells and 0.03% malignant cells were sampled, and the remaining 76 WSIs, which accounted for 62.3% of the whole dataset and included 11 malignant and 65 benign slides, were employed as a separate testing set. Figure 1b shows the data distribution in further detail. For the second experiment, a three-fold cross-validation was performed. During the evaluation, each model was independently assessed on the testing set.

### 3.2. Experimental Settings

The proposed deep learning framework was implemented using the Caffe library on a workstation equipped with four NVIDIA GeForce GTX 1080 Ti GPU cards, Intel Xeon Gold 6134 CPU, and 128 GB memory, and a workstation equipped with one NVIDIA GeForce GTX 1080 Ti GPU card, Intel Xeon CPU E5-2650 v2, and 32 GB memory. For training, the proposed method was trained with the settings as follows: learning rate, $1\times 10^{-10}$; dropout ratio, 0.5; weight decay, 0.0005; and batch size, 1. A stochastic gradient descent (SGD) optimizer was utilized for the optimization, and the categorical cross-entropy function was employed as a loss function. In addition, the baseline models (U-Net, SegNet, and FCN) were implemented utilizing the keras implementation of the image segmentation models by Gupta et al. [44] on the workstation equipped with one NVIDIA GeForce GTX 1080 Ti GPU card, Intel Xeon CPU E5-2650 v2, and 32 GB memory. For training, the baseline models were trained using the default settings based on the codes provided in the literature. Furthermore, for WSI processing, the proposed deep learning model and the baseline models utilized the same framework that is described in detail in Section 2.1.

### 3.3. Evaluation Metrics

The quantitative evaluation of the segmentation performance was produced using four measurements, i.e., precision, sensitivity, Dice similarity coefficient (*DSC*), and Intersection over Union (*IoU*). The evaluation metrics were computed as follows:

(6) $$Precision=\frac{TP}{TP+FP}$$

(7) $$Sensitivity=\frac{TP}{TP+FN}$$

(8) $$DSC=\frac{2TP}{2TP+FP+FN}$$

(9) $$IoU=\frac{TP}{TP+FN+FP}$$

where *TP* denotes the true positives, *TN* represents the true negatives, *FP* denotes false positives, and *FN* is the false negatives.

### 3.4. Quantitative Evaluation with Statistical Analysis

**Figure 5:**
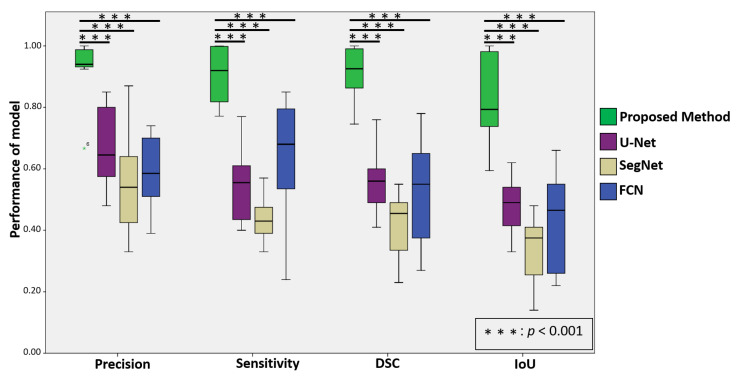
The box plot of quantitative evaluation results of metastatic lesion segmentation where the outliers >3 × interquartile range are marked with an asterisk. The LSD test results demonstrates that the proposed method performs significantly better than the baseline methods (*p*<0.001).

For the first experiment, we evaluated the performance of the proposed method with the three state-of-the-art deep learning models, including U-Net, SegNet, and FCN, as shown in Table 2(a). The experimental results demonstrated that the proposed method achieved a precision of 93.4%, sensitivity of 89.8%, DSC of 82.2%, and IoU of 83.2% while the U-Net model obtained a precision of 67.0%, sensitivity of 54.1%, DSC of 55.7%, and IoU of 47.3%, the SegNet model obtained a precision of 55.8%, sensitivity of 43.8%, DSC of 42.2%, and IoU of 33.6%, and the FCN model obtained a precision of 59.0%, sensitivity of 63.0%, DSC of 52.5%, and IoU of 42.7%, respectively. To further validate the robustness and effectiveness of the proposed method, we assessed the quantitative scores with Fisher’s least significant difference (LSD), using SPSS software [45] (see Table 2(b)). The LSD test results demonstrated that the proposed method significantly outperformed the baseline methods (U-Net, SegNet, and FCN) based on precision, sensitivity, DSC, and IoU (*p*<0.001). Figure 5 presents the box plots of the quantitative evaluation results of the proposed method and three baseline methods (U-Net, SegNet, and FCN) for metastatic lesion segmentation in EBUS-TBNA, showing that the proposed method outperforms the baseline methods in terms of precision, sensitivity, DSC, and IoU (*p*<0.001).

**Figure 6:**
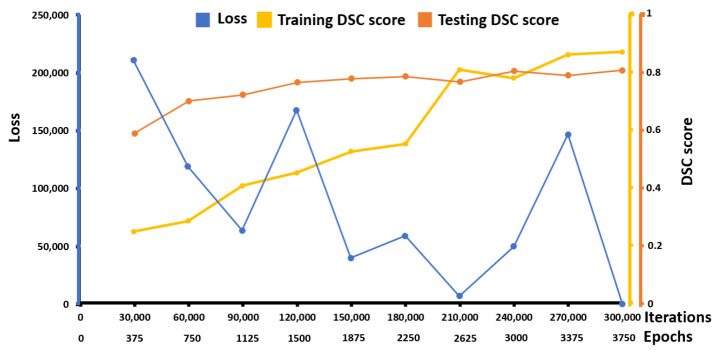
The results of the training loss, the training DSC scores, and the testing DSC scores through iterations/epochs represented in blue and orange, respectively.

For the second experiment using a three-fold cross validation (see Table 3), the proposed method demonstrated a promising performance, higher than the two best performing baseline methods, including U-Net and FCN, with a precision of 91.8 ± 1.2, sensitivity of 96.3 ± 0.8, DSC of 94.0 ± 1.0, and IoU of 88.7 ± 1.8 while the U-Net model obtained a precision of 68.6 ± 17.0, sensitivity of 52.3 ± 9.2, DSC of 57.6 ± 2.8, and IOU of 40.5 ± 2.8 and the FCN model obtained a precision of 72.3 ± 19.0, sensitivity of 59.0 ± 15.4, DSC of 61.6 ± 0.5, and IOU of 44.5 ± 0.6, respectively. The experimental results of the two experiments demonstrated that the proposed method could be used for the automatic detection and segmentation of lymph node metastasis in EBUS-TBNA cytological slides robustly. To further investigate if there was any overfitting or underfitting, Figure 6 shows an epochwise train–test curve that presents the training loss with the training and the testing DSC scores through iterations/epochs, indicating that the proposed model is least likely to be underfitting or overfitting. Figure 6 was generated based on the result of the second fold in the cross-validation.

**Figure 7:**
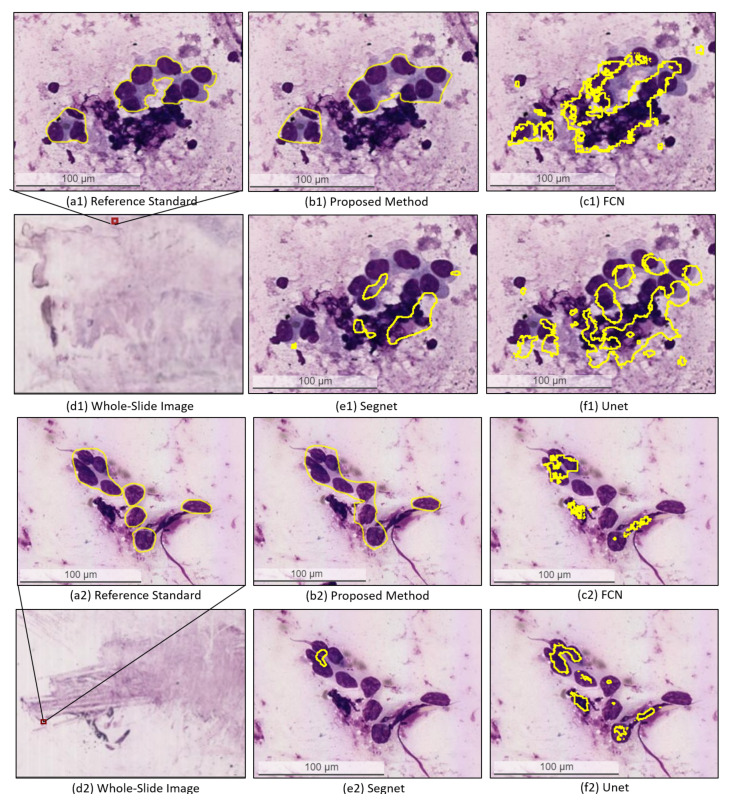
Visual comparison of the typical segmentation results generated by the proposed method and the three baseline methods (U-Net, SegNet, and FCN) for metastatic lesion segmentation in EBUS-TBNA WSIs.

**Figure 8:**
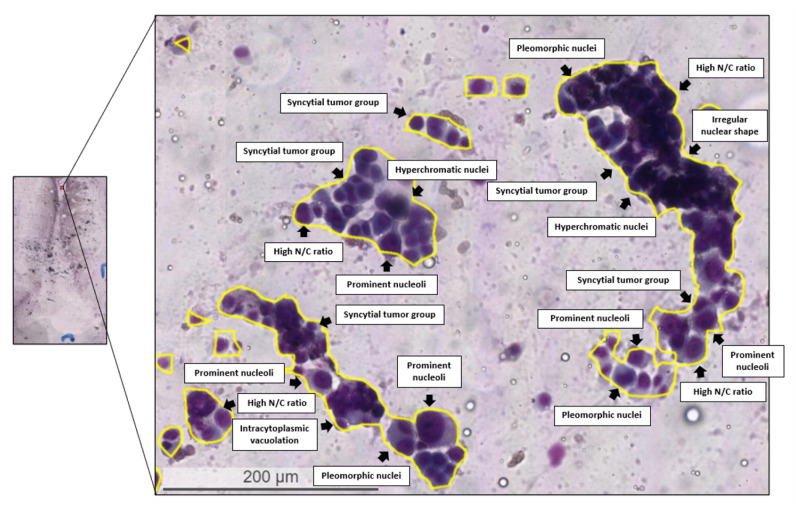
Pathologist’s assessment of automatic predictive results generated by the proposed method with typical lung adenocarcinoma features required to generate a diagnostic evaluation, including syncytial tumor group, hyperchromatic nuclei, high N/C ratio, prominent nucleoli, irregular nuclear shape, pleomorphic nuclei, and intracytoplasmic vacuolation.

For the qualitative evaluation, Figure 7 compares the segmentation outputs by the proposed method and the three baseline methods (U-Net, SegNet, and FCN) for metastatic lesion segmentation. The results show that the proposed method generated annotations consistent with the reference standard produced by an expert pathologist while the three baseline methods performed poorly for metastatic lesions segmentation. Figure 8 presents a pathologist’s assessment of the segmentation results produced by the proposed approach. It can be seen that the automatic predictive results generated by the proposed method demonstrate the typical lung adenocarcinoma features, including syncytial tumor group, hyperchromatic nuclei, high N/C ratio, prominent nucleoli, irregular nuclear shape, pleomorphic nuclei, and intracytoplasmic vacuolation, that are required by pathologists to make a diagnostic evaluation.

### 3.5. Computational Time Comparison

The computational time is critical for methods to be implemented in practical clinical use. To demonstrate the computational efficiency of the proposed method, we analyzed the AI computational time utilizing different hardware specifications (see Section 3.2). We evaluated the computational efficiency of the proposed method and the three baseline methods by calculating the AI inference time on a 77,688 × 41,014 sized WSI. To process a 77,688 × 41,014 sized WSI, the proposed method took less than a minute utilizing four NVIDIA Geforce GTX 1080 Ti GPU cards and 3.6 min using a single NVIDIA Geforce GTX 1080 Ti GPU card while the U-Net model required 9.1 min, the SegNet model required 8.4 min, and the FCN model required 12.4 min, as shown in Table 4. The computational evaluation experiments demonstrated that the proposed technique could process WSI 2.5 times faster than U-Net, 2.3 times faster than SegNet, and 3.4 times quicker than FCN, even with a less expensive GPU, demonstrating that the proposed method was an effective, suitable, and cost-effective solution for processing multigigapixel histopathological images in clinical practice, where rapid diagnosis analyses are required.

## 4. Discussion and Conclusions

The diagnosis of mediastinal metastatic lesions is required to determine the best treatment plan. It is critical for lung cancer staging to correctly classify lymph nodes as benign or malignant. The detection of mediastinal lymph node metastases can save unnecessary procedures. Although TBNA, transesophageal-ultrasound-guided needle aspiration, computed-tomography-guided transbronchial aspiration, and mediastinoscopy can all be used to sample lymph nodes, the majority of these techniques have drawbacks, including the need for general anesthesia, a low yield, and poor accessibility. The EBUS-TBNA has emerged as a revolutionary method to estimate both benign or metastatic lymph nodes with a high diagnostic yield. It has several advantages including being a minimally invasive approach, safe, cost effective, and offering real-time image guidance [11]. In 19% of patients, EBUS-TBNA may give diagnostic information as well as staging information, reducing the need for additional invasive procedures [8]. The EBUS-TBNA may be utilized for on-site assessment because of immediate smears from needle aspiration and brushing specimens [11,12,13,14,15], and the diagnostic rate at various sites ranges from 50% to 90% [46,47,48,49,50,51,52,53,54,55]. The large variation in the diagnosis rate of different centers is due to the manual diagnosis. In this work, we presented a deep learning method that produced consistent and accurate diagnosis of mediastinal metastasis lesions for lung cancer staging. Apart from lung cancer staging, EBUS-TBNA has been used in various medical applications. The accuracy of EBUS-TBNA for detecting mediastinal metastases from extrathoracic malignancy and lymphoma was 85–95 percent [56,57] and 91–97 percent [58], respectively, while the accuracy of EBUS-TBNA for diagnosing sarcoidosis was 79 percent [59]. Our future plans include applying the proposed deep-learning-based framework to other applications such as detecting mediastinal metastasis from extrathoracic malignancy and lymphoma, diagnosing sarcoidosis, detecting endoscopic-ultrasonography-steered fine-needle aspiration for the diagnosis of pancreaticobiliary lesions, and analyzing pleural effusion or ascites. In addition, we are interested in exploring further model improvement, such as incorporating upsampling or the transpose details used for dilation in deconvolution.

Glass slides may now be converted into WSIs, allowing pathological images to be examined using computer-based approaches [22]. WSIs are obtained at very high resolution (order of 10 gigapixels) and are difficult to visually scrutinize thoroughly. Over the last decade, there has been a surge of interest in creating computational tools to assist medical practitioners with improving the efficiency of medical image analysis. There are compelling grounds to assume that digital pathology in conjunction with AI is a viable solution to this problem since it assists the establishment of more exact diagnoses, decreases examination time, and reduces the labor of pathologists as well as the examination cost. The computational cost is a substantial barrier to using computational approaches to diagnose gigapixel WSIs, and as a result, many existing algorithms are not suitable for real-world deployment. A complete and comprehensive automated inspection of WSIs with high accuracy may require additional time and computer resources. We presented in this study an efficient and effective approach for automatic detection and segmentation of lymph nodes metastasis in EBUS-TBNA cytological slides in seconds. Although it is challenging for physicians to detect metastatic lesions using PET/CT imaging, AI utilizing deep learning has shown acceptable and fast diagnostic abilities for distinguishing malignant from benign mediastinal lymph nodes. The effectiveness and robustness of the proposed method was evaluated using two experiments. The experimental results demonstrated that the proposed method achieved promising performance in the segmentation of enlarged mediastinal lymph nodes metastasis in EBUS-TBNA WSIs, significantly outperforming the state-of-the-art baseline approaches (*p*<0.001). The high precision and recall in the experimental results demonstrated that the proposed method could aid the pathologists with aspects of their evaluation applicable to an automatic analysis.

## Figures and Tables

**Table 1:** The architecture of the proposed deep learning network.

Layer	Features (Train)	Features (Inference)	Kernel Size	Stride	Padding
Input	512 × 512 × 3	512 × 512 × 3	-	-	-
Conv1_1 + relu1_1	710 × 710 × 64	710 × 710 × 64	3 × 3	1	same
Conv1_2 + relu1_2	710 × 710 × 64	710 × 710 × 64	3 × 3	1	same
Pool1	355 × 355 × 64	355 × 355 × 64	2 × 2	2	valid
Conv2_1 + relu2_1	355 × 355 × 128	355 × 355 × 128	3 × 3	1	same
Conv2_2 + relu2_2	355 × 355 × 128	355 × 355 × 128	3 × 3	1	same
Pool2	178 × 178 × 128	178 × 178 × 128	2 × 2	2	valid
Conv3_1 + relu3_1	178 × 178 × 256	178 × 178 × 256	3 × 3	1	same
Conv3_2 + relu3_2	178 × 178 × 256	178 × 178 × 256	3 × 3	1	same
Conv3_3 + relu3_3	178 × 178 × 256	178 × 178 × 256	3 × 3	1	same
Pool3	89 × 89 × 256	89 × 89 × 256	2 × 2	2	valid
Conv4_1 + relu4_1	89 × 89 × 512	89 × 89 × 512	3 × 3	1	same
Conv4_2 + relu4_2	89 × 89 × 512	89 × 89 × 512	3 × 3	1	same
Conv4_3 + relu4_3	89 × 89 × 512	89 × 89 × 512	3 × 3	1	same
Pool4	45 × 45 × 512	45 × 45 × 512	2 × 2	2	valid
Conv5_1 + relu5_1	45 × 45 × 512	45 × 45 × 512	3 × 3	1	same
Conv5_2 + relu5_2	45 × 45 × 512	45 × 45 × 512	3 × 3	1	same
Conv5_3 + relu5_3	45 × 45 × 512	45 × 45 × 512	3 × 3	1	same
Pool5	23 × 23 × 512	23 × 23 × 512	2 × 2	2	valid
Conv6 + relu6 + Drop6	17 × 17 × 4096	17 × 17 × 4096	7 × 7	1	same
Conv7 + relu7 + Drop7	17 × 17 × 4096	17 × 17 × 4096	1 × 1	1	same
Conv8	17 × 17 × 3	17 × 17 × 3	1 × 1	1	same
Deconv9	576 × 576 × 3	576 × 576 × 3	64 × 64	32	same
Cropping	512 × 512 × 3	512 × 512 × 3	-	-	-
Softmax	512 × 512 × 3	512 × 512 × 3	-	-	-
Output Class Map	512 × 512 × 1	512 × 512 × 1	-	-	-

**Table 2:** The first experiment: quantitative evaluation and statistical analysis of the proposed method and the baseline methods in metastatic lesions segmentation on EBUS-TBNA WSIs.

(a): Quantitative Segmentation Results							
	**Method**		**Score**			**95% C.I. for Mean**	
			**Mean**	**Std. Deviation**	**Std. Error**	**Lower Bound**	**Upper Bound**
Precision	Proposed Method		**0.934**	0.094	0.028	0.871	0.997
	U-Net [31]		0.670	0.123	0.036	0.592	0.748
	SegNet [32]		0.558	0.158	0.046	0.644	0.656
	FCN [33]		0.590	0.118	0.034	0.515	0.664
Sensitivity	Proposed Method		**0.898**	0.093	0.028	0.836	0.960
	U-Net [31]		0.541	0.114	0.033	0.469	0.613
	SegNet [32]		0.438	0.063	0.018	0.397	0.478
	FCN [33]		0.630	0.202	0.058	0.501	0.758
DSC	Proposed Method		**0.822**	0.283	0.085	0.632	1.012
	U-Net [31]		0.556	0.096	0.277	0.495	0.617
	SegNet [32]		0.421	0.102	0.296	0.356	0.486
	FCN [33]		0.525	0.160	0.046	0.422	0.627
IoU	Proposed Method		**0.832**	0.142	0.042	0.736	0.927
	U-Net [31]		0.473	0.090	0.026	0.416	0.530
	SegNet [32]		0.335	0.106	0.030	0.268	0.403
	FCN [33]		0.427	0.155	0.044	0.328	0.526
**(b): LSD Multiple Comparisons**							
**Dependent Variable**	**(I) Method**	**(J) Method**	**Mean Difference (I−J)**	**Std. Error**	**Sig.**	**95% C.I.**	
						**Lower Bound**	**Upper Bound**
Precision	Proposed Method	U-Net [31]	0.263 *	0.536	<0.001	0.154	0.511
		SegNet [32]	0.377 *	0.536	<0.001	0.268	0.483
		FCN [33]	0.343 *	0.525	<0.001	0.237	0.449
Sensitivity	Proposed Method	U-Net [31]	0.357 *	0.038	<0.001	0.278	0.278
		SegNet [32]	0.460 *	0.386	<0.001	0.382	0.332
		FCN [33]	0.268 *	0.541	<0.001	0.158	0.377
DSC	Proposed Method	U-Net [31]	0.265 *	0.074	<0.001	0.113	0.416
		SegNet [32]	0.400 *	0.074	<0.001	0.248	0.551
		FCN [33]	0.296 *	0.072	<0.001	0.150	0.443
IoU	Proposed Method	U-Net [31]	0.358 *	0.047	<0.001	0.261	0.455
		SegNet [32]	0.496 *	0.047	<0.001	0.399	0.593
		FCN [33]	0.404 *	0.0525	<0.001	0.298	0.510

* The proposed method is significantly better than the baseline methods (U-Net, SegNet, and FCN) using LSD test (*p*<0.001).

**Table 3:** The second experiment: quantitative evaluation of the proposed method and the two best-performing baseline models using three-fold cross-validation.

Model	Fold	Precision	Sensitivity	DSC	IoU
	Fold1	**0.909**	**0.954**	**0.931**	**0.871**
Proposed Method	Fold2	**0.935**	**0.974**	**0.954**	**0.913**
	Fold3	**0.909**	**0.96**	**0.934**	**0.877**
	Mean ± std	**0.918 ± 0.012**	**0.963 ± 0.008**	**0.940 ± 0.010**	**0.887 ± 0.018**
	Fold1	0.560	0.550	0.560	0.385
U-Net [31]	Fold2	0.880	0.420	0.560	0.392
	Fold3	0.620	0.600	0.610	0.438
	Mean ± std	0.686 ± 0.170	0.523 ± 0.092	0.576 ± 0.028	0.405 ± 0.028
	Fold1	0.730	0.550	0.620	0.449
FCN [33]	Fold2	0.910	0.460	0.610	0.438
	Fold3	0.530	0.760	0.620	0.449
	Mean ± std	0.723 ± 0.190	0.590 ± 0.154	0.616 ± 0.005	0.445 ± 0.006

**Table 4:** Computational time comparison of the proposed method and the baseline methods.

Method	WSI Size (pixels)	AI Inference Time (min)
Proposed Method	77,688 × 41,014	**0.9**
Proposed Method	77,688 × 41,014	**3.6**
U-Net [31]	77,688 × 41,014	9.1
SegNet [32]	77,688 × 41,014	8.4
FCN [33]	77,688 × 41,014	12.4

## Data Availability

The data that support the findings of this study are available from the corresponding author upon reasonable request.
